# Harnessing the untapped potential of food education in schools: Nurturing the school subject Food and Health

**DOI:** 10.1111/mcn.13521

**Published:** 2023-04-28

**Authors:** Tormod Bjørkkjær, Päivi Palojoki, Cecilie Beinert

**Affiliations:** ^1^ Department of Nutrition and Public Health, Faculty of Health and Sport Sciences University of Agder Kristiansand Norway; ^2^ Centre for Lifecourse Nutrition University of Agder Kristiansand Norway; ^3^ Department of Food and Nutrition, and Sports Science, Faculty of Education University of Gothenburg Gothenburg Sweden; ^4^ Department of Education, Faculty of Educational Sciences University of Helsinki Helsinki Finland

**Keywords:** adolescents, children, Food and Health education, food education, public health, schools, sustainability

## Abstract

Essential *life skills* related to food and meals have a potential triple dividend for children and adolescents, that is, short‐term, medium‐term and possible generational effects with regard to public health, sustainability and well‐being of future citizens in local communities. While parents and childhood environments are a basis for learning about food and meals, systematic food education in the setting of primary and lower secondary schools may have a significant role that should be utilized more strongly, reaching and benefitting all pupils from a life course perspective. Through this article, we explore the current state of the art of the mandatory school subject Food and Health (FH) from the Nordic perspective. Our leading questions are: (1) What potential is currently utilized and which future potential does FH education have in primary and secondary schools in terms of food education for essential *life skills* and competencies, and (2) How can this untapped potential be better harnessed with a goal of facilitating better learning in FH? Drawing on data from Norway as a case study, supported by Swedish and Finnish data, we discuss the status, challenges and potential reformation of food education, focusing on FH. This includes perspectives on the prioritization of the FH subject and the organization of more systematic food education in schools, which might improve FH's status and significance. Combining theory–practice, creating room for discussion and focusing less on cooking‐related activities may better facilitate learning in FH. Without proper FH, food education might be nonsystematic, thereby generating unequal outcomes for children and adolescents.

## Key messages

- The Food and Health (FH) subject offers enormous learning potential concerning food as it relates to public health, sustainability and well‐being.
- Formal teacher qualification and prioritization of FH as a subject are needed for harnessing its potential.
- Less focus on cooking, a better combination of theory–practice and more time for discussion and reflection may facilitate higher learning levels in FH.
- Schools may be a good setting for systematic food education, given proper FH curricular guidance.

## Graphical abstract

Food and Health (FH) subject in school is an important setting for systematic food education. FH subject should be reformed to utilize its' potential better, to facilitate better learning for pupils in primary and lower secondary schools.

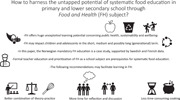

**Abbreviations**
FH: Food and Health
WHO: World Health Organization

## 1 SOCIETAL CHALLENGES REGARDING FOOD AND LEARNING ABOUT FOOD

The importance of, and interest in food, food choice and food environments has grown rapidly in recent decades, especially due to the impact of diet quality on public health, as well as on the environment and climate change (United Nations Environment Programme, 2016). An unhealthy diet is one of the leading causes of ill health and early deaths globally (Afshin et al., 2019) and more sustainable food systems are urgently needed to tackle climate change and reach the United Nations' sustainable development goals (European Commission, Directorate‐General for Research and Innovation, Group of Chief Scientific Advisors, 2020). These global challenges require sufficient action to foster sustainable and healthy lifestyles among people (United Nations Environment Programme, 2016), and there is a need for informed and conscious consumers to practice healthy and sustainable food habits in their daily lives.

Essential *life skills* are particularly relevant for children and adolescents, who possibly more than ever need to adjust their food choices and consider various ethical issues related to food choice and food production (Höglund, 2020). According to the World Health Organization (WHO, 2003), *life skills* can be divided into three areas which are relevant for young populations; (1) communication and interpersonal skills, (2) critical thinking, analysis and decision making and (3) self‐efficacy and the ability to influence and guide one's own life. But where and how are these *life skills* related to food and meals to be learned?

Most children and young people learn at least some basic food preparation skills and eating habits at home (Murcott, 2014; Wolfson et al., 2017). Adolescent cooking skills are positively associated with stronger family connections (Utter et al., 2016), although the importance of family meals may vary in different families (Quarmby & Dagkas, 2015). Socioeconomic differences in food intake are well known (e.g., Fismen et al., 2016), which may give both vulnerable and otherwise healthy children and adolescents better or worse opportunities for good health. Additionally, changes in everyday life and the demands of work life have reduced food preparation in many homes (Gatley et al., 2014), which might be the reason why there are fewer opportunities for young people to learn from their parents. Parents not only influence their children's eating habits through their own food choices, schedules and dining practices but also by sharing their knowledge. It is often thought that children eat what their parents offer. Another aspect is that parents offer what children agree to eat (Williams, 2011).

The everyday environments of children and young people expand with age. Twenty‐first century children and adolescents are exposed to diverse food environments at home, school, and in local communities. While parents are mainly responsible for establishing health‐promoting food habits for their children/adolescents, primary and secondary schools may have a significant role as more formal learning environments in relation to food and meals, as well as preparing pupils with essential knowledge, skills and attitudes.

## 2 FOOD AND HEALTH (FH)—A NORDIC CASE OF A MANDATORY SCHOOL SUBJECT WITH LIFE SKILLS POTENTIAL

The historic and traditional school subject FH is the smallest school subject in primary and lower secondary schools in Norway. The potential of FH for learning about food and meals is especially interesting as it is a mandatory school subject reaching all pupils in primary and lower secondary school, unlike in most European countries and elsewhere in the world. Sweden and Finland also have similar mandatory food education subjects in school, referred to as Home and Consumer Studies in Sweden and Home Economics in Finland. This paper will use Norway and FH education as a case study, supported by research from Sweden and Finland as the subjects share many similarities, possibilities and challenges concerning education and research. In the following text, we, therefore, use the term FH when talking about mandatory food education subjects in Norway, Sweden and Finland.

In this perspective paper, our leading questions are: (1) What potential is currently utilized and which future potential does FH education have in primary and secondary schools in terms of food education for essential *life skills* and competencies, and (2) How can this untapped potential be better harnessed with a goal of facilitating better learning in FH? In our view, as will be elaborated on later in this paper, the latest research suggests that FH should be reformed to have a stronger role in schools and simultaneously be challenged to renew its pedagogical practice to reach its full potential (Beinert, 2021). Concurrently with this reformation, a critical question remains; how can this potential be better harnessed? We will focus on the status, challenges and possible reformation of the FH subject, with a special focus on the competence levels of teachers, prioritization and organization of the subject, and how to facilitate better learning with a theory–practice combination, by creating room for discussion and with less focus on cooking time‐consuming recipes.

## 3 WHAT IS FOOD‐RELATED LEARNING AND FOOD EDUCATION?

Food‐related learning may be seen as an outcome of food education provided in the context of various school subjects, such as FH.

The components of food education are multidisciplinary, since at its broadest definition, food education involves themes such as healthy food and eating, societal and cultural factors and issues of inclusion and sustainability (Smith et al., 2022). This broad variety of content areas can be seen in the current Norwegian FH curriculum throughout its core elements; *healthy diets*, *sustainable food habits and consumption*, and *food and meals as an expression of identity and culture* (Ministry of Education and Research, 2019), in addition to the interdisciplinary topics *public health and life skills* and *sustainable development* (Ministry of Education and Research, 2017).

Since the determinants related to food, eating and health are multifaceted, it is interesting to look at the various views presented in previous studies on food education more closely. The content areas of ‘food education’ can be itemized into at least four overlapping themes: (i) food education in the context of health education and nutrition (e.g., Contento, 2011), (ii) in relation to sustainability and safety (e.g., Swan & Flowers, 2015), (iii) in relation to food‐based concepts (e.g., S. Fordyce‐Voorham, 2011; Pendergast & Dewhurst, 2012) and (iv) in relation to social, cultural, economic and environmental aspects (e.g., Kimura, 2011).

The outcomes of food education also vary. For example, food education can be seen as a means of learning ‘food skills’, referring to skills in food selection, procurement, preparation and eating (Porter et al., 2000). These skills present themselves as increased personal knowledge, personal competencies and as the ability to use available resources and seek information (S. Fordyce‐Voorham, 2011; S. P. Fordyce‐Voorham, 2016). Aiming for ‘food‐related citizenship’ means that the young can understand the role and functioning of sustainable food systems (Wilkins, 2005), thereby obtaining skills which perform on a societal level. They learn also to acknowledge the rights and duties citizens have regarding food (Lozano‐Cabedo & Gómez‐Benito, 2017) and food systems (Rico Mendez et al., 2021). Food agency, defined as the interaction between individual choices and social structures (Lahne et al., 2017; Trubek et al., 2017; Wolfson et al., 2017) can also be seen as an outcome of more socially oriented food education. This abundant area of knowledge is indeed a challenge for teachers and curriculum developers, but at the same time the potential of food education and FH education is based on this rich variety; there are many interesting aspects to add to the national curricula, spanning from individual perspectives to societal levels. Still, many of the aforementioned outcomes of food education are already present through competence aims in the Norwegian FH curriculum (Ministry of Education and Research, 2019).

Food education in the national curricula should always be research‐based and follow the current nutritional recommendations of the country in question, as in Norway (Ministry of Education and Research, 2019). The development of sustainable eating habits, those which align with nutritional recommendations promoting public health, can be supported with the help of well‐planned and well‐contextualized food education. Such an example, in line with systematic food education, can potentially support learners to understand the effects of their food choices and activities on their own or others' well‐being (see Gisslevik et al., 2019; Janhonen et al., 2016). ‘Understanding the effects’ is a key phrase here. In a world full of food‐related messages, it is not self‐evident that young people understand complex relations and have the critical thinking skills to filter out nonsense or fake information regarding food. This leads us to a discussion of the qualities of food education. In our view, well‐planned food education is especially important in contemporary society as a wide range of both true and false food‐related information is available, and messages provided by real or fake experts spread quickly on social media platforms favoured by young people (Pilgrim & Bohnet‐Joschko, 2019; Qutteina et al., 2022).

### 3.1 Schools as an important setting for systematic food education

School education can be viewed as a strategic public health approach directed on a structural level, as education is a highly modifiable determinant of health (The Lancet Public Health, 2020), giving all age groups the possibility to acquire essential skills and competencies. This is especially relevant for disadvantaged groups, and in this respect, systematic food education through proper FH might possibly rectify unequal social conditions for upcoming generations. Such a life course perspective of early efforts may yield a substantial triple dividend for children and young adults in the short, medium (Black et al., 2022; Hanson & Aagaard‐Hansen, 2021) and possibly long (generational) term. Indeed, Warwick‐Booth and Aggleton (2021) and Hargreaves et al. (2022) highlight school as an important setting for health and nutrition education, and food education is important in the 21st century according to Kostanjevec and Lovšin Kozina (2021).

In countries where the FH subject does not have an established role in school systems, such as in most central European countries and elsewhere in the world, important *life skills* connected with food need to be covered by parents or caregivers who may or may not have such knowledge, skills and competencies. Recently, with the Covid‐19 pandemic and school shutdowns, we have seen increases in obesity and social inequality among children (Jenssen et al., 2021), and Pendergast (2021) points out the potential and importance of food education in this context. Wistoft et al. (2021) found that around 20% of pupils felt lost, with inadequate support during homeschooling during the initial Covid‐19 lockdown in Denmark. Interestingly, Lichtenstein and Ludwig (2010) called for more food education in American schools over 10 years ago, thereby acknowledging the importance of FH education. They argue that essential skills related to food may be lost in future generations if there is not sufficient support from the school system. More recently, McCloat and Caraher (2020) highlighted the need for mandatory food education as part of school curricula. Ballam (2018, 2021) also revisits the importance of taking food education in the UK seriously in recent editorials.

## 4 STATUS, CHALLENGES AND POTENTIAL REFORMATION OF THE SCHOOL SUBJECT FH

### 4.1 Teacher's formal competence, school organization and prioritization of FH

In Norway, alarmingly, less than half of the FH teachers have a formal teaching qualification in the subject, which is the lowest proportion of qualified teachers across all school subjects (Arnesen et al., 2022; Vik et al., 2020). Studies even indicate that FH teachers view their experiences as home cooks, and food as a personal interest, as being significant for their competence in FH (Holthe et al., 2013). Currently, only those working in lower secondary schools from the 8th to the 10th grades need 30‐course credits or ECTS (one full semester) in FH from their teacher training to teach the subject (Ministry of Education and Research, 2015). For those teaching FH in lower grades, there are no requirements regarding formal competence. Furthermore, FH classes from the 1st to the 4th grades have not been prioritized at all despite curriculum goals in place after 4th grade. If FH classes are allocated in the span of the 1st to the 4th grades, it seems arbitrary as to which teacher is assigned to teach, as teachers' interest in the subject and who functions as the head teacher for the class are the decisive factors of who teaches the subject rather than competence (Helland et al., 2021). This is a major school organization issue which highlights the current low status of FH in the Norwegian school system.

Research has shown how a larger proportion of qualified teachers in FH translates to teachers which are more content and which experience a larger degree of mastery in their FH teaching compared with their colleagues without formal competence (Vik et al., 2020). One practical example is linked to digital skills and the dilemma of choosing quality‐assured teaching material, and knowing how to guide pupils through the internet jungle of ‘experts’ or ‘so‐called experts’, as illustrated in LEAD and NAVI‐HED projects (University of Helsinki, 2022). Another example is how pupils can challenge the teacher and influence the pedagogy surrounding food choices, as discussed by Bohm et al. (2015, 2016) in Sweden concerning attitudes towards meat and its necessary inclusion in a ‘proper meal’, as opposed to a primarily vegetarian option.

Although there is a focus on offering further education to increase the number of teachers with competence in the subjects they already teach, it has also been suggested to require all teachers, including those working from the 1st to the 7th grade, to have FH competence to teach the subject in Norway as a strategy to strengthen the food and nutrition competence in the public sector (Torheim et al., 2020). The new government in Norway for the period 2021–2025 has a special focus on practical and aesthetical subjects, thereby stating a focus on providing further education for teachers (Prime Minister's Office, 2021). This is both a political responsibility as well as a responsibility found at the local level, with principals or school owners driving this change forward. By doing so, the Norwegian government is following the Finnish teacher education system, which has since the 1970s provided master's level education to all class and subject teachers working at the comprehensive schools (1st–10th grade) (Ministry of Education and Culture, 2016). Due to this, similar challenges with lack of formal competency of FH teachers, do not exist in Finland. In summary, improved competence levels in FH may professionalize the subject and raise its status to a level similar to theoretical subjects such as Mathematics. For example, this reformation could start with a stronger focus on using *subject terms* (Lassen, 2020) and not letting one's own perceptions and experiences be the basis for the lessons (Veka et al., 2018, 2020). Interestingly, Veka et al. (2020) found that FH teachers are often working alone, that is, with a limited professional community, and little influence from the headmaster. Indeed, the FH curriculum is competence‐based, as opposed to content‐based or teacher‐based. And although the choice of pedagogical approach is a teacher's prerogative, choosing the most suitable approach is assumedly easier with a minimum level of subject‐specific competence.

### 4.2 Popularity of practical teaching approaches in FH

Practical aesthetical subjects are popular among pupils, which studies from Norway (Beinert, Palojoki, et al., 2020; Holthe et al., 2013), Sweden (Skolverket, 2004) and Finland (Paas & Palojoki, 2019) confirm. Teachers included in the study conducted by Holthe et al. (2013) expressed how they viewed FH lessons as a break for the pupils in an otherwise theoretical school day. This may be a warning sign; if competence aims are not acknowledged, the lessons could lose status as worthwhile knowledge‐building activities. Findings from Beinert, Palojoki, et al. (2020) also reveal that both pupils and teachers value practical work in the act of cooking during FH classes. Still, teachers express a desire and need to include more theoretically oriented food and nutrition education but find this difficult because of the limited time available (Beinert, Palojoki, et al., 2020).

The FH subject has gone through several reforms since its conception; however, the tradition of cooking has been consistently maintained over the years. Practical work related to food and meals is still at the core when teachers plan FH lessons. This focus is so strong that Veka et al. (2018) call the recipes used the ‘hidden curriculum’, meaning the recipe is the central focus when planning and conducting the lesson, not the competence aim in the curriculum. This might explain why FH teachers in Norway believe they contribute more to pupils' practical cooking skills than developing their knowledge about healthy diets (Bottolfs, 2020). As Beinert et al. (2022) propose, the FH subject should have a stronger impact on pupils' food choices, linking it to their current and future lives in accordance with the WHO *life skills* framework (WHO, 2003) and life course perspective (Black et al., 2022; Hanson & Aagaard‐Hansen, 2021). At present, most knowledge about food and sustainable eating comes from the home or media, whereas schools provide only a small fraction of information on the subject (Beinert, 2021; Höijer, 2013). This might be partially explained by the fact that FH is the smallest subject in the Norwegian, Swedish and Finnish schools and because cooking‐related activities are prioritized, as previously described. In addition, Bohm (2022a) describes how putting too much focus on cooking and housework in FH in Sweden may lower the subject's status and marginalize it among the other school subjects.

The FH curriculum in Norway includes several competence aims after Years 4, 7 and 10. What the pupils are expected to be able to do after the 10th grade is listed in Box 1.

Box 1 **Overview of competence aims after 10th grade**

**Table:** 

plan and use suitable utensils, techniques and cooking methods to create safe and sustainable food that lays the foundation for good health use their senses to assess the quality of foods, explore and combine flavours in cooking and improve recipes, menus and food preparation discuss how diet may contribute to good health, and use digital resources to assess their own diet and to choose healthy and a variety of different foods when cooking describe and critically assess claims, advice and information about diet and health critically assess information about food production and discuss how consumer power can impact local and global food production explore the carbon footprint of foods, and describe how food choices and food consumption may impact the environment, climate and food safety make food from Norway, Samiland and other cultures, and compare and explore raw ingredients and cooking methods used in different food cultures demonstrate how cooking and meals convey identity and community in different cultures Source: Ministry of Education and Research (2019)

These competence aims focus on many aspects that go beyond practical cooking skills, but as stated, they receive less attention in FH classes. Given the limited hours allocated to FH, typically 2–3 school hours per session, planning the content and learning achievements within each session in accordance with the yearly plan, is of utmost importance. By replacing some of the time spent cooking, and by preparing fewer and simpler dishes connected to learning activities directed at, for example, understanding food in relation to health and sustainability, it may be possible to maximize the learning outcomes of FH lessons through a broadening of the pupils' perspectives. This will also emphasize the competencies related to critical thinking, discussing, and planning as stated in the curriculum. Therefore, focusing on reforming the subject to consist of a more comprehensive approach to food education has been argued for (Beinert, 2021). Although the current FH curriculum opens for such teaching approaches and learning opportunities, cooking is still a strong part of the competence aims, and traditions dictate how the FH subject is planned in Sweden and Norway (Bohm, 2022a, 2022b; Holthe et al., 2013). Given that around 80% of the time in FH is used for practical cooking (Beinert, Øverby, et al., 2020), we question whether lack of time is the reason for the insufficient focus on other activities, or if the time available can be spent more wisely from a learning perspective. This latter issue is very interesting regarding the nature of the subject and also acknowledges teachers' experiences of the shortage of time available (Beinert, Palojoki, et al., 2020; Bohm, 2022b). As Bohm (2022b) notes, FH teachers can never close the book and say, ‘We'll continue next week’. The key question is how the time available is used in the best pedagogical way and focused on the pupils' learning as opposed to the teachers' teaching. Bohm (2022b) refers to the term ‘time poverty’; that is, complex and vague curriculum content being taught, together with cultural expectations and scheduling issues, mixed with pupils' cooking that may not always be timed well. As such, instead of pupils reflecting on themes such as sustainability in classes, they work in groups with time‐consuming recipes and cleaning, meaning there is pupil activity but possibly unexploited learning opportunities. Is it indeed time to critically look at what is done in FH classes; the kitchens where teaching takes place can be used for other learning activities, not only cooking sessions, or the pupils can receive instruction in regular classrooms when they are not actively preparing food. Food‐related learning can also be organized outside traditional classrooms, such as outdoors or in collaboration with local resources, potentially expanding the learning space in FH lessons.

A recent study investigated *lesson signature* in FH, that is, observation of lesson organization, time allocation, type of teaching or learning forms, and how the different parts of the lessons were eventually connected (Aadland & Wergedahl, 2022). Though a high activity level was observed among pupils in the above study, time was mainly spent on group tasks related to practical kitchen work. Little time was spent on listening to peers (and/or the teacher). Discussions, even those taking place between pupils, were basically absent. It thus seems that social learning is not as pronounced as one often assumes with a practical subject like FH. According to the study by Aadland and Wergedahl (2022), one typically begins with teacher‐led instruction, followed by group activities and eating. In our view, such a teaching approach needs to be adjusted to achieve the less directly cooking‐related competence aims. However, as the data from the above study was derived from teaching the previous curriculum in FH in Norway, these findings need to be confirmed with newer data derived after the current curriculum (Ministry of Education and Research, 2019) was formally introduced in autumn 2020.

### 4.3 Combining theory and practice and facilitating pupils' learning processes through discussion

There are huge pedagogical challenges regarding food‐related learning in schools. But at the same time, there is also a lot of pedagogical potential. If FH lessons are planned in a way that ‘the theory’ (e.g., the main public health and climate challenges) remains too distanced from ‘the practice’ (e.g., food preparation or everyday food choices), the understanding of nutrition‐related concepts may remain fragmented, inconsistent and mixed with unscientific beliefs. This may be due to incomplete or erroneous factual knowledge internalized from social media, or from subjective efforts to explain complex issues (e.g., heart disease) through more familiar, yet simplifying concepts (e.g., ‘good’ or ‘bad cholesterol’). But doing all this in a pedagogically smart way reverses the outcome completely. Theoretical and school‐learned concepts can be better transferred into the practical context of everyday life, thereby becoming more easily understandable, when the learning tasks at school are pupil‐centred and practical (Beinert, 2021), or when the theoretical concepts are operationalized in *food preparation* tasks during FH lessons. Bohm et al. (2016) also highlight theory versus practice as a critical issue in FH. This places even more emphasis on the quality of practical food preparation and cooking during FH lessons: if used wisely, these practical elements may support the understanding of the more theoretical concepts and issues of the lessons. If the pedagogical point is missing, then we are talking about ‘over‐cooking’, which is the time‐consuming preparation of food to be eaten as opposed to using food as a tool for learning. Importantly, pupils may potentially be more engaged, empowered, and motivated if they are given age‐appropriate space and time to express themselves more instead of being mainly teacher‐led (Aadland & Wergedahl, 2022; Veka et al., 2018).

One additional way of achieving higher learning levels (e.g., analysing and not just remembering or understanding) in the FH classrooms may be through *interthinking*, which is a form of dialogue that aids pupils in cooperative meaning‐making and supports their critical thinking (Taar & Palojoki, 2022; Vass et al., 2014). In an action research study focused on interthinking in FH classrooms, Taar and Palojoki (2022) showed how FH pupils achieved joint thinking episodes in parts of FH lessons which are in line with 21st century skills such as critical thinking, collaboration, communication and creativity (Partnership for 21st‐century learning, 2019). In joint thinking processes the pupils are in a social decision‐making process, helping each other, arguing, asking questions, demonstrating reason and explaining and justifying their reasons for their thinking. In the above case of interthinking, the *why* of the learning process is more evident, which may seem to be less focal in FH classes where the *what* and *how* of cooking and cleaning is pronounced, as stated earlier (Bohm, 2022a, 2022b; Höijer, 2013). Achieving this level of higher learning also depends on the pupils and their potential for learning, which may differ from class to class and with age. Newer studies on *lesson signature* in FH described above would yield important data on how much space and time for interthinking is actually created during FH classes.

## 5 OVERALL DISCUSSION AND CONCLUSION

In this article, we have explored the current utilized potential and future possible potential of promoting public health and sustainability through FH education and we have discussed how food education can be reformed to better support a more systematic food‐related learning. Previous studies discussed in this article suggest that the potential of FH is not currently properly utilized. Importantly, variation in teacher competence, variation in how FH lessons are given and the quality of their contents (e.g., not following formal curricula), and how some local school authorities prioritize and organize the FH subject negatively impact the systematic nature of food education. Nationally, the FH subject also has a low status and is allocated the least amount of hours in school, which is intriguing given the huge public health and sustainability issues found in modern society.

From the learner's viewpoint, the key question is how the future potential of FH as part of food education in schools can be better harnessed? How should teachers better support individual learners, and redevelop and reform the contents and pedagogical methods used during the FH lessons? We believe we need to ask ourselves two questions. First, how is the limited time in FH classes used in schools? Is cooking dominating the time used to such an extent that it takes valuable time away from reflecting and discussing what was being done and why during the lesson? How is the ‘theory’ merged with ‘practice’: does the prepared food give new insights and illustrate, for example, the role of various sugars in the diet or how our diet can play a key role in combatting climate change? Or is FH merely about making and eating food? As we have argued here, we should investigate new ways of facilitating learning to achieve the curricular aims, as discussed by Haapaniemi et al. (2022).

Second, are school authorities prioritizing FH according to its potential in primary and lower secondary schools, and is FH properly organized at the school level (e.g., at 4th grade) to the benefit of pupils? We believe local school authorities need to question who should be given the role of FH teacher. Should this choice be dependent on individual teachers' interests in cooking or merely due to their role as the head teacher, or should formal competence matter? By discussing how to take a more systematic approach to food education, and thereby emphasizing each competence aim in the curriculum equally, the FH subject's potential of being a health‐promoting subject in the school may be increased. The three *life skills* defined by the WHO (2003); (1) communication and interpersonal skills, (2) critical thinking, analysis and decision making and (3) self‐efficacy and the ability to influence and guide one's own life can more easily be developed in FH classes. However, the process of developing more systematic food education requires refocusing the contents and learning methods of the FH classes, finding a better balance between theory and practice, time for discussion, and less time spent on cumbersome recipes, and most importantly, trusting the pupil's learning processes under the lead of professionally qualified teachers.

In light of current societal public health and sustainability challenges, increased food prices, and the European war affecting food security on a global level, it is timely to question how the importance of the smallest school subject can be taken more seriously inside and outside of schools. And as such, FH could increase in importance for pupils' learning and future lives, thus harnessing the FH subjects' potential. We, unfortunately, know little about the impact of FH specifically on pupils and their lives per se (Beinert et al., 2022), but we argue that it is plausible it may have an impact with more systematic food education in the short, medium and long term. Is it worth gambling with our future generations? They deserve a proper food education.

While several of the studies referred to in this article are based on data collected in the era of the last Norwegian curricula (Ministry of Education and Research, 2006), one might speculate that the Covid‐19 pandemic has negatively impacted the implementation process. There are indications that at least the process towards interdisciplinary working, as introduced in new Norwegian curricula from 2020 (Ministry of Education and Research, 2019), may seem a bit cumbersome (Biseth et al., 2022). Thus, it is important to investigate in future studies how FH is affected by the content of the new curriculum, and how it will be applied in schools.

In conclusion, we see an enormous untapped potential within the established school subject FH for future food education in schools, given its huge relevance for future public health and sustainability of pupils, community and our globe. Therefore, all efforts, at the governmental level, to the local school authorities and to the individual teachers, are needed to reach the youth in schools to raise their interest concerning the importance of everyday food choices and to motivate them to see the benefits of well‐reasoned food choices, benefitting both their own health and the health of the environment.

## AUTHOR CONTRIBUTIONS

Tormod Bjørkkjær, Päivi Palojoki and Cecilie Beinert designed and conceptualized the paper, and drafted and contributed to writing the paper. All authors critically revised and approved the final manuscript.

## CONFLICT OF INTEREST STATEMENT

The authors declare no conflict of interest.

## Data Availability

No new data were created or analyzed in this perspective article, discussing published articles and white papers. Data sharing is thus not relevant.
